# Multidrug resistance in pathogenic *Escherichia coli* isolates from urinary tract infections in dogs, Spain

**DOI:** 10.3389/fvets.2024.1325072

**Published:** 2024-02-23

**Authors:** Ana Abad-Fau, Eloisa Sevilla, Ainara Oro, Inmaculada Martín-Burriel, Bernardino Moreno, Mariano Morales, Rosa Bolea

**Affiliations:** ^1^Departamento de Patología Animal, Facultad de Veterinaria, Instituto Agroalimentario de Aragón-IA2, Universidad de Zaragoza, Zaragoza, Spain; ^2^Centro de Encefalopatías y Enfermedades Transmisibles Emergentes, Facultad de Veterinaria, Universidad de Zaragoza, Zaragoza, Spain; ^3^Laboratorio de Genética Bioquímica, Facultad de Veterinaria, Instituto Agroalimentario de Aragon, Universidad de Zaragoza, Zaragoza, Spain; ^4^Albéitar Laboratories, Zaragoza, Spain

**Keywords:** dog, *Escherichia coli*, multidrug resistance, urinary tract infection, virulence factors

## Abstract

*Escherichia coli* (*E. coli*) is a pathogen frequently isolated in cases of urinary tract infections (UTIs) in both humans and dogs and evidence exists that dogs are reservoirs for human infections. In addition, *E. coli* is associated to increasing antimicrobial resistance rates. This study focuses on the analysis of antimicrobial resistance and the presence of selected virulence genes in *E. coli* isolates from a Spanish dog population suffering from UTI. This collection of isolates showed an extremely high level of phenotypic resistance to 1st–3rd generation cephalosporins, followed by penicillins, fluoroquinolones and amphenicols. Apart from that, 13.46% of them were considered extended-spectrum beta-lactamase producers. An alarmingly high percentage (71.15%) of multidrug resistant isolates were also detected. There was a good correlation between the antimicrobial resistance genes found and the phenotypic resistance expressed. Most of the isolates were classified as extraintestinal pathogenic *E. coli*, and two others harbored virulence factors related to diarrheagenic pathotypes. A significant relationship between low antibiotic resistance and high virulence factor carriage was found, but the mechanisms behind it are still poorly understood. The detection of high antimicrobial resistance rates to first-choice treatments highlights the need of constant antimicrobial resistance surveillance, as well as continuous revision of therapeutic guidelines for canine UTI to adapt them to changes in antimicrobial resistance patterns.

## 1 Introduction

Urinary tract infections are one the most common causes of primary care veterinary supervision in dogs and a treatment challenge due to their high recurrence and therapeutic implications. *Escherichia coli* is the most common bacterium isolated in UTIs in dogs and humans (1–3). In addition, *E. coli* bacteremia in humans (the most common cause of bacteremia in high-income countries) is caused by urinary tract infections in more than 50% of cases (4). *Escherichia coli* has also been associated with an increase in antimicrobial resistance (2, 5). Evidence suggests that dogs act as a reservoir of human infections with uropathogenic *E. coli* (UPEC) and are a source of spread of antimicrobial resistance (6).

*Escherichia coli* is classified into various pathotypes based on the presence of virulence factors. Uropathogenic *E. coli* is included within the group of extraintestinal pathogenic *E. coli* (ExPEC) and are characterized by specific virulence factor. Some of these virulence factors include P-fimbriae (*papC*), α-haemolysin (*hlyA*) and cytotoxic necrotizing factor type 1 (*cnf1*) (7). *Eae*, the gene that codifies for intimin and is associated with diarrheic strains, can also be found in uropathogenic strains (8). Other relevant *E. coli* virulent factors include Shiga toxins (*Stx*), also known as verotoxins and characteristic of Shiga toxin-producing *E. coli* (STEC), which are related to hemorrhagic diarrhea and hemolytic uremic syndrome, and have been described in several species, although natural infections are rarely described in dogs (9, 10). There is a potential zoonotic risk associated with the presence of these genetic elements in companion animals and other species (11, 12). Hybrid strains have gained recent attention, especially those that harbor several virulence factors traditionally associated with different pathotypes. These new types of strains are considered “heteropathogen” or hybrid, such as STEC/UPEC strains (2), and are considered as able to produce both outcomes, diarrhea, or UTI (13).

Virulence genes are encoded by plasmids, bacteriophages, or pathogenicity islands (PAI). Pathogenicity islands are mobile and unstable fragments of DNA present in pathogenic strains, but absent in the related non-pathogenic strains, which can be shared by horizontal transmission. *PapC*, *hlyA* and *cnf1*, among other virulence genes, are usually encoded simultaneously within PAIs in UPEC (14, 15). P-fimbriae, encoded by *papC* gene, plays an important role in kidney adherence and the inflammatory response (16). α-Haemolysin is a toxin known to produce renal injuries, and even though the mechanism is still unclear, *cnf1* does not play a major role in the severity of the disease but it is usually associated with other virulence genes (15).

It has been previously demonstrated that some canine UPEC isolates are clonal with those isolated from humans, suggesting their zoonotic potential. It has also been proposed that dogs could act as a reservoir of this *E. coli* pathotype, hence the importance of the study of the potential implications of UTI in this animal species (17, 18).

In the last few decades there has been a rising concern about the increase in the number of *E. coli* isolates presenting a multidrug resistant (MDR) profile (19, 20). It has been described that the ownership of companion animals could be a risk factor in the spread of pathogenic *E. coli* strains between humans and pets, also favoring the dissemination of antimicrobial resistance in the community (21–23).

It is common to find antibiotic resistance in *E. coli* isolates from cases of UTI, which highlights the importance of monitoring the strain susceptibility to the antibiotic treatment, even if an experimental treatment has already been implemented. In fact, UPEC strains isolated from dogs have been described as MDR reservoirs in several countries (24, 25) and as carriers of extended-spectrum beta-lactamase (ESBL) genes (5). ESBL-producing *E. coli* have been previously found in cats and dogs, and human-dog co-carriage in the same household has also been demonstrated in fecal samples (26–28). In general, ESBL and the presence of other antibiotic resistance mechanisms can difficult the treatment of infectious diseases and, therefore, result in complicated chronic infections.

Although microbiological culture and susceptibility testing are recommended before any antimicrobial therapy is established, empiric treatment is frequently established and the most common recommendations to treat these infections in companion animals include amoxicillin (without clavulanic acid) and trimethoprim-sulfonamides as a first approach (29). Therefore, updated information about antimicrobial susceptibility patterns is highly needed.

The aim of this study was to determine the presence of *E. coli* in urine samples from a Spanish dog population presenting clinical signs of urinary tract infections and to characterize the isolates according to selected virulence factors and their antimicrobial resistance pattern, a research field scarcely investigated in Spain.

## 2 Materials and methods

### 2.1 Collection of *Escherichia coli* isolates

This study was conducted on a total of 52 *E. coli* isolates. This collection of isolates came from urine samples from dogs diagnosed with UTI. Samples were aseptically collected by cystocentesis as part of the daily activity of private veterinary practitioners in Zaragoza, Spain. The criteria followed to diagnose UTI were those used in everyday clinic, which include frequent urination, pain during urination, fever or vomiting, among others. The sampling period ranged from 2017 to 2019, and all urine samples were taken before any treatment was established. Mean age of the individuals was 8.97 years old (95% CI: 4.11–13.83%). In regard to the gender of these individuals, 21 of them were male and 31 were female.

Isolates were identified using VITEK® (bioMérieux, France) and those confirmed as *E. coli* were then stored at −20°C for further analysis.

### 2.2 Virulence gene detection

DNA was extracted by boiling 3–5 colonies from pure cultures and then conventional PCR for the detection of virulence-related genes was performed. These genes included *eae* (intimin), *Stx1* and *Stx2* (Shiga toxins 1 and 2), *papC* (P-fimbriae), *hlyA* (α-haemolysin) and *cnf1* (cytotoxic necrotizing factor type 1). Primers used in this study were those described in Table 1. PCR was performed in a BiometraTRIO 48 thermocycler (Analytik Jena, Germany), and PCR products were analyzed under UV light in 1.5% agarose gels stained with GelGreen® (Biotium, United States).

**Table 1:** Primers used in conventional PCR performed in this study.

Gene	Primer	Sequence (5′ → 3′)	Annealing temperature (°C)	Amplicon size (bp)	Reference
*eae*	eae-common-F	CCCGAATTCGGCACAAGCATAAGC	55	881	(30)
	eae-common-R	CCCGGATCCGTCTCGCCAGTATTCG			
*Stx1*	EC-vt1_2-F	CGTCTTTACTGATGATTGATAGTGGC	58	637	(31)
	EC-vt1_2-R	CGCGATGCATGATGATGAC			
*Stx2*	EC-vt2_2-F	TACCACTCTGCAACGTGTCG	58	297	(31)
	EC-vt2_2-R	CGATACTCCGGAAGCACATT			
*papC*	pap1	GACGGCTGTACTGCAGGGTGTGGCG	55	328	(32)
	pap2	ATATCCTTTCTGCAGGGATGCAATA			
*hlyA*	hlyA-F	AACAAGGATAAGCACTGTTCTGGCT	55	1,177	(33)
	hlyA-R	ACCATATAAGCGGTCATTCCCGTCA			
*cnf1*	cnf1-A	GAACTTATTAAGGATAGT	54	543	(32)
	cnf1-B	CATTATTTATAACGCTG			

CECT 4783 strain was used as positive control for *eae*, *Stx1* and *Stx2* genes; C136b strain was the positive control for *hlyA* and *cnf1* genes, kindly provided by Dr. J. A. Orden, University Complutense of Madrid, Spain. A canine strain previously isolated by our research group (Pe8 strain, GenBank accession number MK034302) was used as positive control for *papC* gene.

*Escherichia coli* isolates were classified in pathotypes according to the presence of the virulence factor genes analyzed. Enterohemorrhagic *E. coli* (EHEC) are described as those *E. coli* strains that harbor both intimin and Shiga toxins (12). When only one of these virulence factors was present, isolates were classified as enteropathogenic *E. coli* (EPEC) or STEC, respectively. If any of the other virulence factor genes analyzed were found, that is *hlyA, PapC* and/or *cnf1*, isolates were classified as extraintestinal pathogenic *E. coli* (ExPEC) (34).

### 2.3 Antimicrobial susceptibility testing

Susceptibility to 74 different antimicrobials was determined using VITEK® (bioMérieux, France). The antimicrobial agents selected to test each isolate susceptibility depended on VITEK® guidelines, as clinically relevant antimicrobials recommended by VITEK® varied during the period in which the study was performed. Antimicrobials were classified in 12 categories: aminoglycosides, amphenicols, carbapenems, fluoroquinolones, nitrofurans, other β-lactams, penicillins, tetracyclines, sulfonamides, 1st–2nd generation cephalosporins, 3rd generation cephalosporins and 4th–5th generation cephalosporins, as shown in Table 2. All isolates were tested against at least one antibiotic of each category, except for 4th and 5th generation cephalosporins, which were added in the middle of the study. For those isolates having no information regarding 4th–5th generation cephalosporins, neither susceptibility nor resistance was included, and they were thus excluded from the prevalence analysis for that group. Resistance to a category of antimicrobials was defined as resistance to at least one of the agents in that category. MDR isolates were defined as those isolates with non-susceptibility to three or more antimicrobial categories (35).

**Table 2:** Antimicrobials tested in *E. coli* isolates and category classification.

Antimicrobial categories	Antimicrobials included in each category			
Aminoglycosides	Amikacin	Gentamicin	Neomycin	
	Isepamycin	Netilmicin	Tobramycin	
Amphenicols	Chloramphenicol			
Carbapenems	Doripenem	Ertapenem	Imipenem	Meropenem
1st and 2nd generation cephalosporins	Cephalexin	Cephalothin	Cefadroxil	
	Cefradine	Cefaclor	Cefonicid	
	Cefamandole	Cefotiam	Cefuroxime	
	Cefmetazole	Cefotetan	Cefoxitin	
3rd generation cephalosporins	Cefpodoxime	Ceftiofur	Cefsulodin	
	Cefditoren	Cefixime	Cefoperazone	
	Cefotaxime	Ceftazidime	Ceftizoxime	
	Ceftriaxone	Cefoperazone/Sulbactam	Ceftazidime/Avibactam	
	Cefpirome	Cefcapene	Cefdinir	
	Latamoxef	Cefmenoxime	Cefteram	
	Cefovecin			
4th and 5th generation cephalosporins	Cefepime	Cefozopran	Ceftobiprole	Ceftolozan/Tazobactam
Fluoroquinolones	Enrofloxacin		Marbofloxacin	
	Pradofloxacin		Ciprofloxacin	
Nitrofurans	Nitrofurantoin			
Other beta-lactams	Loracarbef	Faropenem	Aztreonam	
Penicillins	Ampicillin	Temocillin	Oxacillin	
	Ampicillin/sulbactam	Carbenicillin	Amoxicillin	
	Amoxicillin/clavulanic acid	Mecillinam	Ticarcillin	
	Ticarcillin/clavulanic acid	Piperacillin	Piperacillin/Tazobactam	
	Azlocillin	Mezlocillin	Benzylpenicillin	
Sulfonamides	Trimethoprim/Sulfamethoxazole			
Tetracyclines	Doxycycline	Tetracycline	Minocycline	

Additionally, VITEK 2 ESBL test (bioMérieux) was used in these isolates for rapid detection of extended-spectrum β-lactamase (ESBL) production, which is based on simultaneous assessment of the inhibitory effects of cefepime, cefotaxime, and ceftazidime, alone and in the presence of clavulanate.

Breakpoints for the interpretation of minimal inhibitory concentration (MIC) results were applied according to the criteria stablished by bioMérieux for small animals (AST-GN97, bioMérieux, France), which include natural resistance and breakpoints from the Clinical and Laboratory Standards Institute (36).

Intermediate resistance category provides a flexible information in clinical practice. However, *E. coli* isolates have been previously found to harbor resistance genes (37) For this reason, when they had to be categorized into dichotomic variants they were assessed as resistant.

### 2.4 Whole genome sequencing

Those isolates showing the highest rate of phenotypic resistance, that is resistance to six or more antimicrobial categories, were selected for further characterization through whole genome sequencing (WGS). A total of ten *E. coli* isolates were cultured for 24 h in Nutrient Agar (Oxoid, United Kingdom) and DNA was then extracted using Wizard Genomic DNA Purification Kit (Promega, Madison, WI, United States). Quality parameters for DNA were checked both on Qubit 4 (Invitrogen) and gel electrophoresis. Genome sequencing was performed on an Illumina Miseq platform with a paired-end read length of 150 bp. Sequences were trimmed on Galaxy (Version 0.3.8.1) and assembled with Unicycler (Galaxy version 0.5.0 + Galaxy 1). All sequencing data have been submitted to NCBI Genome Database under BioProject PRJNA1031085, and individual accession numbers are the following: SAMN37924970 (isolate 258.883), SAMN37926527 (isolate 262.947), SAMN37926528 (isolate 263.715), SAMN37926529 (isolate 266.493), SAMN37926530 (isolate 267.252), SAMN37926531 (isolate 269.901), SAMN37926532 (isolate 271.550), SAMN37926533 (isolate 271.758), SAMN37926534 (isolate 271.811) and SAMN37926535 (isolate 271.960).

Antibiotic resistance genes, virulence factors, serotypes and sequence types (ST) were assigned to these sequenced genomes using tools that included ResFinder 4.1 (38–40), PathogenFinder 1.1 (41), VirulenceFinder 2.0 (39, 42), MLST 2.0 (*E. coli* #1 and #2) (39, 43–48), cgMLSTFinder 1.2 (42, 49), MGE v1.0.3 (39, 50–52) and SeroTypeFinder 2.0 (53). Visualization of the genomic data was carried out using Proksee (54). A phylogenetic tree was created with Roary pipeline (55) based on Prokka annotation (56), and followed by use of IQ-TREE software (57).

### 2.5 Statistical analysis

Prevalence was calculated with 95% confidence intervals (CI). To test simple relationship between virulence factors and antibiotics, Fisher ‘s Exact Test was used, and the *p*-values determined, considering them statistically significant when value of *p* ≤0.05. Numeric values were calculated using Pearson’s coefficient. Isolates showing intermediate antibiotic resistance were considered as resistant for statistical comparisons. All the analyses and calculations were performed using R version 4.1.1 and RCommander 2.7–1.

## 3 Results

### 3.1 Virulence factor analysis

According to the virulence factor analysis performed, the prevalence of the virulence-related genes was as follows: 1.92% for *eae* (95% CI: 0–5.66%), 1.92% for *Stx2* (95% CI: 0–5.66%), 59.62% for *papC* (95% CI: 46.28–72.95%), 53.85% for *hlyA* (95% CI: 40.30–67.4%) and 32.69% for *cnf1* (95% CI: 19.97–45.44%). However, *Stx1* gene was not found in this study.

Regarding *E. coli* pathotype classification, 82.69% (95% CI: 79–87%) of isolates were classified as ExPEC, and around 20% (9/43) of them simultaneously harbored the three extraintestinal virulence factors analyzed. Additionally, 1.92% (95% CI: 0–5.66%) of isolates were defined as EPEC, and the same value was found for STEC. However, no EHEC isolates were detected. None of the virulence factors analyzed in this study were found in 13.46% (95%CI: 3–23%) of the isolates.

### 3.2 Prevalence of phenotypic antimicrobial resistance

According to the antimicrobial resistance profiles observed, only one out of 52 (95% CI: 0–5.66%) *E. coli* isolates was susceptible to all the antimicrobials tested. Also, all the antimicrobial categories presented resistant isolates, although in a variable percentage.

**Figure 1:**
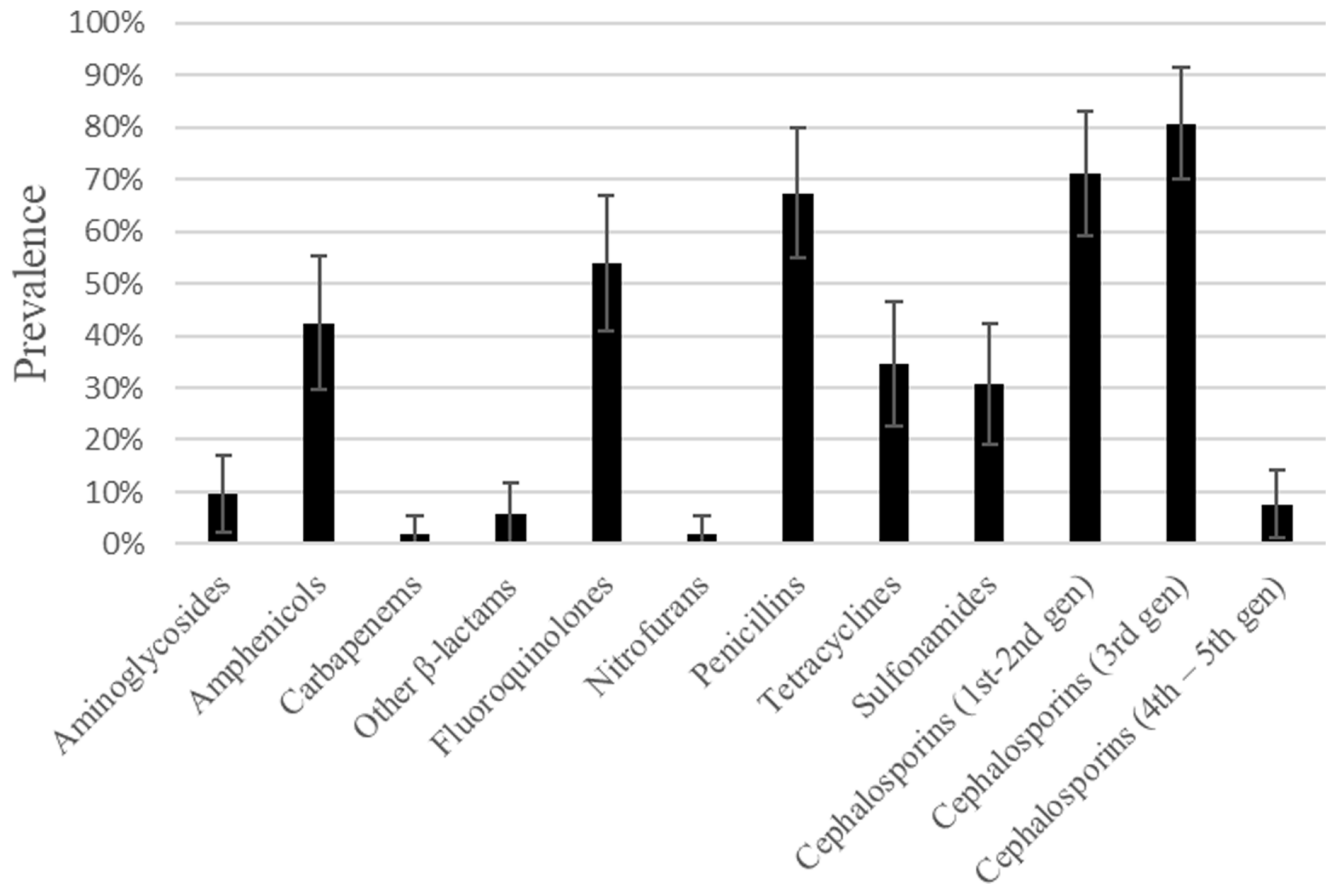
Prevalence of resistance to different antimicrobial categories found in *E. coli* isolates from dog urine.

According to antimicrobial resistance levels defined by the European Food Safety Authority (58), an extremely high resistance level was found for the categories of 1st–2nd and 3rd generation cephalosporins, followed by very high resistance to penicillins and fluoroquinolones. These isolates also displayed a high resistance level to amphenicols (Figure 1). A low resistance level was found in 5 out of the 12 categories: carbapenems, nitrofurans, other β-lactams, 4th–5th generation cephalosporins and aminoglycosides.

Several isolates showed resistance to antibiotics which are considered critically important antimicrobials and are listed in category A (59). For example, three (out of 30) isolates were resistant to the β-lactam aztreonam, and there were several others found resistant to category A antibiotics from the penicillin group: three (out of 17) isolates were resistant to carbenicillin, 18 (out of 31) to ticarcillin, one (out of 33) to piperacillin and three (out of three) to mezlocillin. There was also one isolate showing intermediate resistance, and thus classified as resistant, to an agent from the carbapenem category (imipenem).

Apart from that, 13.46% (95% CI: 4.17–22.73%) of the isolates were considered ESBL-producers, and almost 60% (4/7) of them showed resistance to 9 or more out of the 12 antibiotic categories tested.

### 3.3 Multidrug resistant profiles

A total of 71.15% (95% CI: 58.84–83.46%) of the studied isolates were described as MDR.

Two main profiles of MDR, with a prevalence of 7.69% (95% CI: 0.45–14.93%) each of them, were observed. The isolates included in one of these profiles showed resistance to the following antimicrobial categories: penicillins, 1st–2nd and 3rd generation cephalosporins; while the other profile comprised those isolates resistant to amphenicols, fluoroquinolones, penicillins, and 1st–2nd and 3rd generation cephalosporins.

### 3.4 Genomic analysis of selected isolates

*In silico* molecular typing was performed in the sequenced genomes from those selected phenotypically resistant isolates (Table 3). Three different nomenclatures for sequence typing were assigned to each isolate according to Achtman’s MLST scheme, Pasteur MLST scheme and core genome (cg)-MLST.

**Table 3:** Sequence types (ST) and serogroups of sequenced *E. coli* isolates.

Isolate ID	Serogroup	ST (Achtman)	ST (Pasteur)	cgMLST
267.252	O15H1	393	494	163,945
258.883	O5H20	6,448	901	174,146
271.960	O4H31	372	490	135,819
262.947	O5H20	6,448	901	174,146
263.715	O5H20	6,448	901	174,146
266.493	O9aH30	224	479	143,321
269.901	O9H17	88	74	11,260
271.550	O8H25	58	24	207,634
271.758	O8H9	90	66	202,038
271.811	O183H18	117	48	187,123

**Figure 2:**
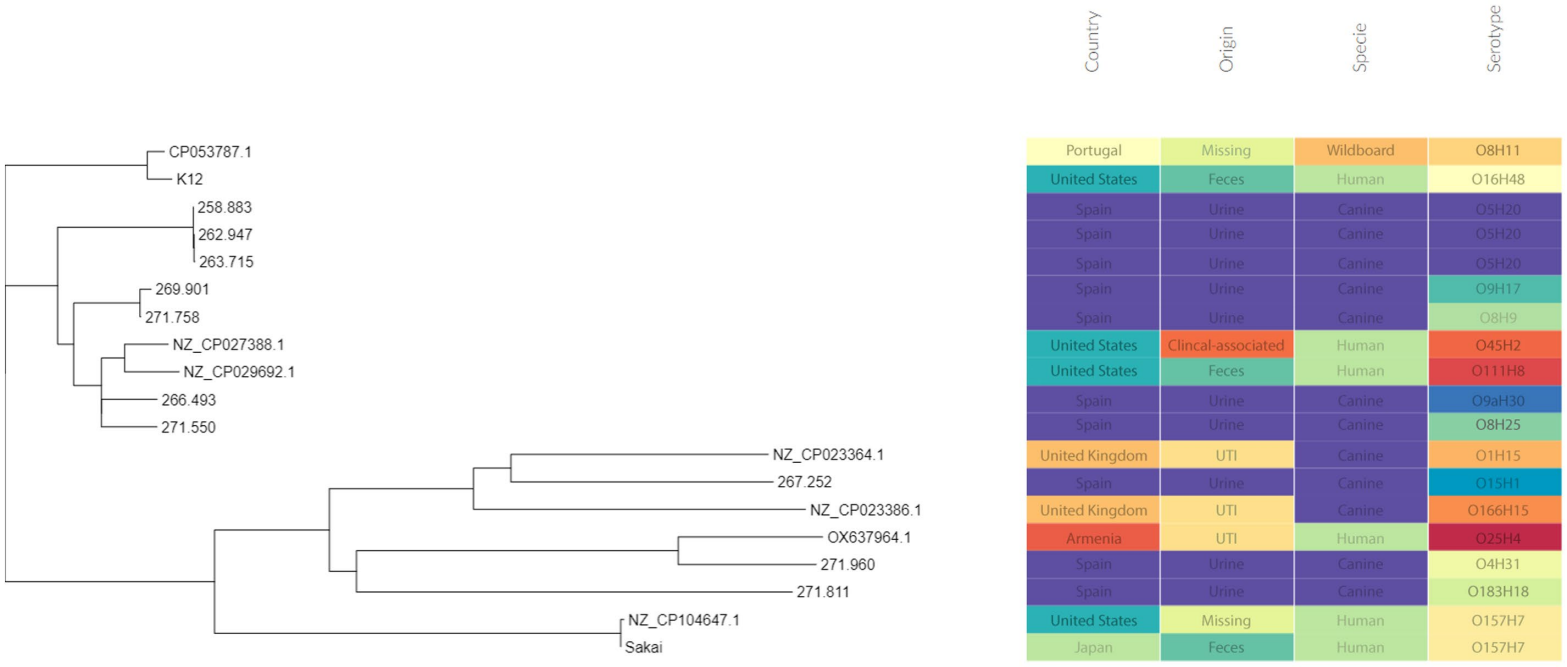
Phylogenetic tree including the sequenced *E. coli* isolates. Metadata was added using Phandango web application (60).

**Figure 3:**
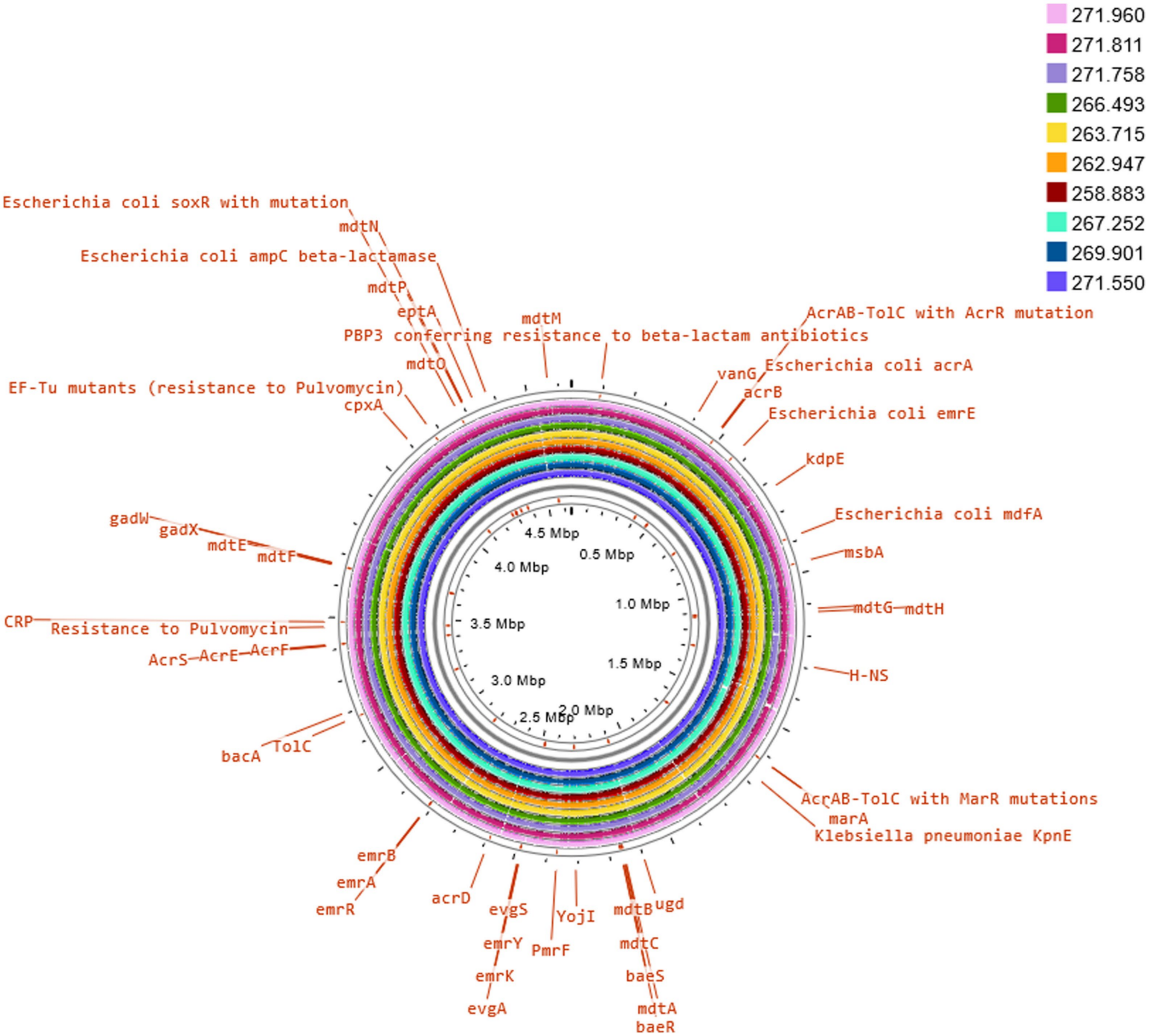
Comparison of *E. coli* K12 reference genome with sequenced isolates. The annotation of selected antimicrobial resistance genes was carried out on Proksee Server from the Stothard Research Group (University of Alberta, Canada) that uses BLAST analysis to illustrate conserved and missing genomic sequences (available online: https://proksee.ca/).

Three of these isolates, that is 258.883, 262.947 and 263.715, shared the same serotype (O5H20), and the corresponding sequence type (Pasteur ST 901 / Achtman ST 6448) and core genome sequence type (cg-ST 174146), making this *E. coli* type the most prevalent one among the studied isolates. The rest of the isolates presented unique molecular types, although isolates 271.758 and 269.901 belonged to the same clonal complex (CC ST23) and were paired together in the phylogenetic tree (Figure 2). Annotated comparison of the isolates (Figure 3) showed no major missing regions.

Several genes and mutations associated with resistance to different antimicrobial categories were detected in the sequenced isolates and are detailed in Table 4. In addition, some of these genes were associated to mobile genetic elements (MGE), which are described in Table 5.

**Table 4:** Antimicrobial resistance genes and mutations found in the sequenced genomes of the selected *E. coli* isolates, as well as the corresponding phenotypic resistance pattern showed in the susceptibility testing assay.

Isolate ID	Aminoglycosides	Phenicols	Beta-lactams	Fluoroquinolones	Tetracyclines	Sulfonamides	Other categories	Phenotypic resistance
258.883	*aph*(6)-Id, *aph*(3″)-Ib, *aadA5*	*floR*	*bla*_CTX-M-55_	parC:p.S80I, gyrA:p.S83L, gyrA:p.D87N	*tet*(A)	*dfrA17*		A-AN-FL-P-S-CP
262.947	*aadA1*, *aadA2b*, *aadA5*	*cmlA1*	*bla*_CTX-M-55_, *bla*_TEM-1B_	parC:p.S80I, gyrA:p.S83L, gyrA:p.D87N	*tet*(A)	*dfrA17*, *sul2*, *sul3*	*qacL*	AN-B-FL-P–T-S-CP
263.715	*aph*(6)-Id, *aph*(3″)-Ib, *aadA5*	*florR*	*bla*_CTX-M-55_, *bla*_Z_	parC:p.S80I, gyrA:p.S83L, gyrA:p.D87N	*tet*(A), *tet*(M)	*dfrA17*		AN-B-FL-P–T-S-CP
266.493	*aadA5*			parC:p.S80I, gyrA:p.S83L, gyrA:p.D87N, parE:p.S458A		*dfrA17*, *sul1*	*erm(B)*, *mph(A)*, *qacE*, *sitABCD*	AN-C-FL-N-P–T-S-CP
267.252	*aph*(6)-Id, *aph*(3″)-Ib		*bla*_TEM-1B_	parC:p.S80I, gyrA:p.S83L, gyrA:p.D87N, parC:p.S57T, parE:p.L416F	*tet*(B)	*sul2*	*sitABCD*	AN-B-FL-P–T-S-CP
269.901	*aadA1*, *ant*(2″)-Ia, *16S_rrsC:g.926_926del*	*floR, catA1*	*bla*_OXA-1_	parC:p.S80I, gyrA:p.S83L,gyrA:p.D87N, parE:p.S458A	*tet*(B)	*dfrA36*, *sul 1*, *sul2*	*qacE*	A-AN-FL-P–T-S-CP
271.550	*aph*(6)-Id, *aph*(3″)-Ib, *aph*(3′)-Ia		*bla*_TEM-1B_	gyrA:p.S83L	*tet*(A)	*dfrA5*, *sul2*	*sitABCD*	A-FL-P–T-S-CP
271.758				parC:p.S80I, gyrA:p.S83L, gyrA:p.D87N, parE:p.S458A				AN-FL-P–T-CP
271.811	*aph*(6)-Id, *aph*(3″)-Ib		*bla*_TEM-1B_		*tet*(B)	*dfrA8*, *sul2*	*sitABCD*	AN-FL-P–T-S-CP
271.960	*aph*(6)-Id, *aph*(3″)-Ib, *aadA1*	*cat86, catA1*		parC:p.S80I, gyrA:p.S83L, gyrA:p.D87G, parE:p.I355T	*tet*(A)	*dfrA1*, *sul1*, *sul2*	*qacE*, *sitABCD*	AN-FL-T-S-CP

A, Aminoglycosides; AN, Amphenicols; C, Carbapenems; B, Beta-lactams; FL, Fluoroquinolones; N, Nitrofurans; P, Penicillins; T, Tetracyclines; S, Sulfonamides; CP, Cephalosporins.

**Table 5:** Presence of mobile genetic elements (MGE) in sequenced isolates, and antibiotic resistance and associated virulence genes.

Isolate ID	MGE	Type	Coverage (%)	Identity (%)	Associated resistance and virulence genes
258.883	IS*Ec9*	IS	100	100	*bla*_CTX-M-55_
	MITEEc1	MIR	100	98.88	*terC, yehB, yehD, yehA, yehC*
	IS*Ec1*	IS	99.77	96.74	*fdeC*
	IS*26*	IS	100	100	*-*
	IS*421*	IS	99.78	99.7	*-*
262.947	IS*Vsa3*	IS	100	100	*sul2*
	IS*Ec9*	IS	100	100	*bla*_CTX-M-55_ *, terC*
	MITEEc1	IS	100	97.56	*yehB, yehD, yehA, yehC*
	IncFIC	PL	100	100	*traT, anr*
	IS*102*	IS	100	92.72	*cma, cba*
	IS*Ec1*	IS	99.77	96.74	*fdeC*
	IS*640*	IS	99.91	98.36	*-*
	IS*421*	IS	99.78	99.7	*-*
263.715	IS*Ec9*	IS	100	100	*bla*_CTX-M-55_
	Tn*6009*	ICE	100	99.89	*tet*(M)
	MITEEc1	MIR	100	97.56	*terC, yehB, yehD, yehA, yehC*
	IS*Ec1*	IS	99.77	96.74	*fdeC*
	IS*421*	IS	99.78	99.7	*-*
	IS*26*	IS	100	100	*-*
266.493	IS*6100*	IS	100	100	*mph(A), qacE, dfrA17, sul1, aadA5*
	MITEEc1	MIR	100	100	*terC, nlpl, terC, yehB, yehD, yehA, yehC, csgA, hlyE*
	IS*5*	IS	100	99.75	*irp2, gad, fyuA*
	IncFII	PL	98.85	95.06	*traT*
	IncFIB	PL	100	98.93	*-*
	IncFIA	PL	100	99.74	*-*
	IncX1	PL	100	94.92	*-*
267.252	IncQ1	PL	65.83	100	*aph*(6)-Id*, aph*(3″)*, sul2*
	IncFII	PL	99.62	96.95	*anr*
	IncFIB	PL	100	99.22	*-*
	IncX4	PL	100	98.88	*-*
	IncFIA	PL	100	99.74	*-*
	IS*Ec45*	IS	100	99.86	*iucC, papA, papC, iutA, sat, iha*
	IS*Ec46*	IS	100	99.94	*fyuA, irp2*
	IS*Ec1*	IS	100	98.06	*csgA, ompT*
	MITEEc1	MIR	99.19	97.56	*terC*
269.901	Incl1	PL	100	100	*cia*
	IS*Ec78*	IS	99.84	98.97	*fyuA, irp2*
	MITEEc1	MIR	100	98.37	*yehD, iss, fdeC*
271.550	IncQ1	PL	65.83	100	*aph*(6)-Id*, aph*(3″)*, sul2*
	IncFIB	PL	100	98.39	*cia, iroN, iss, mchF, etsC, cvaC, etsC, ompT, hlyF*
	IncFII	PL	100	100	*traT, anr*
	IS*Ec31*	IS	99.28	92.73	*terC*
	MITEEc1	MIR	99.19	94.26	*iss, fdeC, terC*
	IS*Ec38*	IS	100	94.6	*fyuA, irp2*
271.758	IncFII	PL	100	100	*-*
	IncFIA	PL	100	99.74	*-*
	IS*3*	IS	100	99.92	*hlyE, csgA*
	MITEEc1	MIR	100	97.56	*yehB, yehD, yehA, yehC, terC, nlpl*
	IS*Ec1*	IS	100	97.91	*fdeC*
271.811	IncFII	PL	100	100	*sul2, aph*(6)-Id*, aph*(3″)-Ib*, anr*
	Incl1	PL	100	100	*-*
	Col(MG828)	PL	98.85	95.38	*-*
271.960	IncQ1	PL	66.46	100	*dfrA1, aadA1, aph*(6)-Id*, qacE, tet*(A)*, aph*(3″)-Ib*, sul1, dfrA1, catA1, sul2, dfrA1*
	IncFII	PL	98.85	98.05	*traT, anr, traJ*
	IncHI2A	PL	100	99.52	*-*
	IncHI2	PL	100	100	*-*
	IS*Kpn37*	IS	99.68	97.3	*hlyA, cnf1*
	MITEEc1	MIR	100	100	*terC*
	IS*Ec38*	IS	99.94	97.16	*cea*

PL, plasmid; IS, insertion sequence; MIR, miniature inverted repeat; ICE, Integrative Conjugative Element.

**Figure 4:**
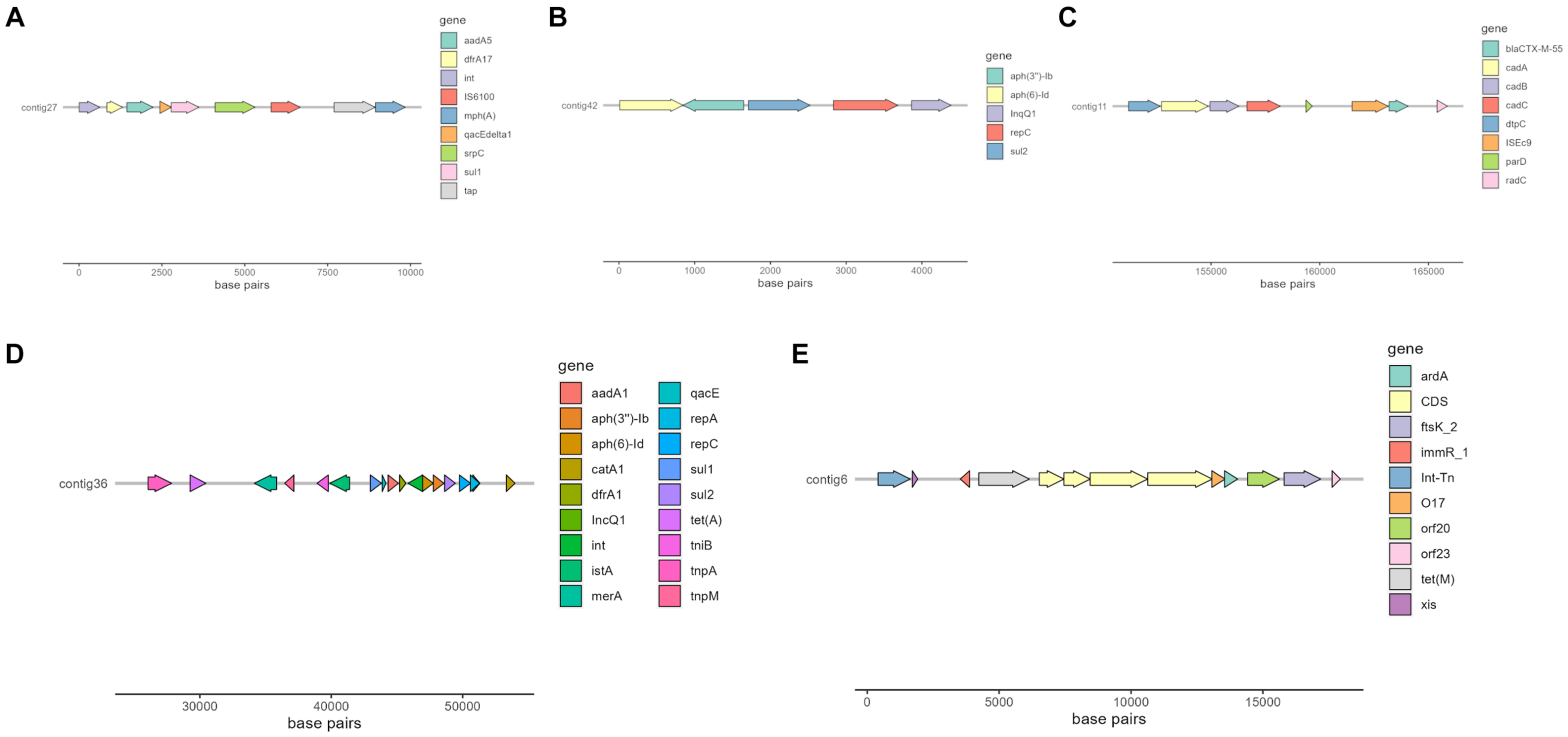
Selected contigs of different isolates containing antimicrobial resistance genes as well as MGE annotated with Prokka (56). **(A)** Organization of a fragment of contig 27 in isolate 266.493, which contains *aadA5*, *dfrA17*, *qacE*, *sul1*, and *mphA* genes. **(B)** Organization of contig 42 in isolate 269.901, which contains *aph*(6)-Id, *aph*(3″)-Ib and *sul2* genes. **(C)** Organization of a fragment of contig 11 in isolate 258.883, which contains *bla*_CTX-M-55_ gene. **(D)** Organization of a fragment of contig 36 in isolate 271.960, which contains several resistance genes, such as *aadA1*, *sul2* and *tet*(A). **(E)** Organization of a fragment of isolate 263.715, which contains *tet*(M) resistance gene.

Interestingly, there were two MGE of particular interest due to its association with important resistance genes or a high number of them, that is IS*6100* and IS*Ec9*, which can be seen in Figure 4.

### 3.5 Association between antimicrobial resistance and virulence factors

When testing for simple relationships between phenotypic antimicrobial resistance and presence of virulence factors in these isolates, the carriage of *cnf1* gene showed a significant association with resistance to several penicillins, as well as with the penicillin category itself (Value of *p* = 0.01). *Cnf1* gene also showed a significant association (Value of *p* = 0.019) with MDR category. Apart from that, age of individuals was significantly associated with *E. coli* isolates showing resistance to various cephalosporins and to the 4th–5th generation cephalosporin category (Value of *p* = 0.034). Gender was associated with aminoglycoside resistant isolates (Value of *p* = 0.007).

ESBL production showed association with resistance to five out of the 12 antimicrobial categories tested (amphenicols, other β-lactams, 4th–5th generation cephalosporins, sulfonamides and fluoroquinolones). No significant relationship between the number of virulence-related genes and ESBL production was observed, however a negative relationship between the number of antimicrobial categories to which isolates showed resistance and number of virulence-related genes was found (Pearson coefficient = 0.33 value of – = 0.014).

## 4 Discussion

This study has evaluated both phenotypic and genotypic antimicrobial resistance as well as the presence of selected virulence genes in *E. coli* isolates from Spanish dogs with UTI and has shown that dogs may be reservoirs of resistant uropathogenic strains of *E. coli*.

Comparing with results obtained in a previous study (61) in which samples were collected in a similar time and geographical location, although they had a different origin (feces), we found that *E. coli* isolates from urine were more susceptible to aminoglycosides than those obtained from dog feces (phenotypic resistance found in 9.62% vs. 40% of isolates). In general, the prevalence of antimicrobial resistances found are similar to those found in *E. coli* isolated from UTI patients (62, 63), except for the penicillin group, which was slightly higher in this study (67.31% vs. ~ 45–50%). Such high prevalence is also contrary to the decreasing trend of penicillin resistance in *E. coli* isolates in Europe (20). Some hypotheses for this phenomenon could be the trend of increase of antibiotic resistance year to year or the fact that more antibiotics from the penicillin group were studied in this work.

*Escherichia coli* aminoglycoside resistance was the only type of resistance linked to the gender of the animal, being found exclusively in male individuals. In fact, male gender has previously been associated with aminoglycoside resistance in Gram-negative bacteria (64).

When taking into account some of the antibiotics considered clinically important for human and animal health by the European Medicines Agency (59), it is worth mentioning that *E. coli* isolates showing resistance to several of these antibiotics were detected in this study, even to antibiotics from category A (“Avoid,” it includes antibiotics not authorized in veterinary medicine in the European Union), such as certain penicillins or carbapenems. Another relevant antibiotic category is category B (“Restrict”), which includes those listed as highest priority critically important antimicrobials (HP-CIAs) by the World Health Organization categorization, e.g., 3rd generation cephalosporins or fluoroquinolones. Indeed, as much as 80.77 and 53.85% of these isolates were considered resistant to 3rd generation cephalosporins and fluoroquinolones, respectively. The high amount of overall resistance found among all the categories could be biased by the fact that complicated UTI are more often requested for culture and antibiogram testing than simpler cases of UTI.

The treatment with amoxicillin or trimethoprim-sulfonamides as first-line agents is currently recommended for the management of bacterial UTI in dogs (29). However, considering these results, it seems that the use of these antimicrobials may be ineffective in a high percentage of cases, since 58.33% of isolates were found to be resistant to amoxicillin and 30.77% to trimethoprim-sulfonamides. Before suggesting any change in current guidelines for antibiotic treatment in canine UTI, it should be noted that *E. coli* is not the only pathogen responsible for UTI and that the data analyzed in this study might be overestimating the baseline resistance, mostly because of the selection of the patients. In any case, the use of antibiotic resistance testing as a routine allows not only the monitoring of the epidemiology of antibiotic resistance profiles but also the faster implementation of a treatment in case of failure of the empiric one.

It is worth mentioning the high percentage of MDR isolates found (71.15%). Among the MDR isolates found, more than 80% (30/37) were classified as ExPEC, and one of them corresponded to EPEC pathotype. This kind of strains possesses a potential zoonotic risk and can also serve as a reservoir of resistance genes (18), further contributing to the dissemination of antibiotic resistance and limiting the options for the treatment of infectious diseases in both humans and animals.

High antibiotic resistance has been associated with MGE in *Enterobacteriaceae*. Bacteria harboring these mobile elements can become a reservoir for antibiotic resistances and be transmitted then from pets to their owners or the environment. This phenomenon poses a serious health problem due to the spread of resistance and failure of current antibiotic treatments (65).

Of special interest is the presence of transposon Tn6009 carrying *tet*(M) gene in isolate 263.715. This element has been previously described in other Gram-negative bacteria, such as *Enterococcus fecalis* (66), and is associated with tetracycline resistance due to the presence of *tet*(M) (Figure 4E). This non-composite conjugative transposon is of clinical importance in Gram-positive bacteria and has a potential role in the dissemination of resistance (67, 68). Resistance to beta-lactams was encoded by *bla*_CTX–M–55_ gene in three isolates, which was located in a IS*Ec9* insertion sequence (IS*1380-like*). IS*Ec9* region has been previously associated with ESBL genes (69) and has been described in other bacteria such as *Vibrio vulnificus* (70). In all cases, the gene and the insertion sequence were 46 bp away. One of them (isolate 263.715) also harbored a *bla*_Z_ gene, and another one (isolate 263.715) had a copy of *bla*_TEM-1B_. Beta-lactamase-encoding genes such as *bla*_TEM-1B_ and *bla*_OXA-1_ were also identified in other isolates. For example, *bla*_TEM-1B_ was present in two isolates that displayed no phenotypic resistance to beta-lactams (isolates 271.550 and 271.811), and in one that did (isolate 267.252). There was only one isolate (269.901) containing a *bla*_OXA-1_ gene. As expected, this isolate was resistant to several antibiotics in the penicillin group (ampicillin, amoxicillin + clavulanic acid, amoxicillin), and it was negative in the ESBL production test *Bla*_OXA-1_ has been found in ST131 or associated with other genes in plasmids (71). Despite this gene being originally described in MGE (72), we only identified IncI1 plasmid in this isolate, and it was not associated with any antibiotic resistance gene. Sulfonamide resistance genes (*sul 1* or *2*) were found in most of the sequenced genomes (7/10), and in five of them these genes were located in a MGE (IS*6100* and IncQ1 for *sul1*, and IS*Vsa3*, IncQ1 or IncFII for *sul2*). All these MGE also harbored other resistance genes, including those linked to streptomycin (*aadA5*, *aph*(6)-Id, *aph*(3″)-Ib *and aadA1* in IS*6100,* IncFII and IncQ1) (73–76), trimethoprim (*dfrA17* and *dfrA1* in IS*6100* and IncQ1) (77, 78), antiseptics (*qacL* and *qacE* in IS*6100* and IncQ1, although all antiseptic resistance genes were incomplete) (79), erythromycin (*mph*(A) in IS*6100*) (80), doxycycline and tetracycline (*tet*(A) in IncQ1) (81) and chloramphenicol (*catA1* in IncQ1) (82).

The IS*Vsa3* transposase was found in one of the sequenced isolates (262.947) and contained the *sul2* gene, which has also been identified in other enteropathogens (83–85). Isolate 266.493 harbored *dfrA17*-*aadA5*-*qacEdelta1*-IS*6100*-*mph*(A)-*sul1* integron structure, which is commonly identified in ExPEC pathotype (86).

Regarding the plasmids identified, IncFII plasmid has been previously documented in Spain as frequently linked to ESBL production (87). In this study, the plasmid was only identified in one isolate, although it was classified as non-ESBL producer.

Another plasmid identified was IncQ1, commonly found in *E. coli* and with ability to transfer between different bacterial species and strains, which facilitates the dissemination of antibiotic resistance in bacterial populations (88). This plasmid was detected in the genomes of three isolates (267.252, 271.550 and 271.960) and was found close to resistance genes linked to aminoglycoside resistance (*aph(6)-Id* and *aph(3″)-Ib*). All these three isolates were distant in the phylogenetic tree, which suggests that the plasmid has been likely acquired independently. One of these isolates harbored nine more resistance genes close to the detected plasmid (Figure 4D), indicating a potential hotspot for antibiotic resistance dissemination.

When studying antibiotic resistance genes in sequenced isolates, there was in general a consistent correlation between phenotypic and genetic resistance. However, there were two significant exceptions to this pattern. When examining aminoglycosides, several isolates exhibited susceptibility to this category despite carrying resistance genes related to both streptomycin and spectinomycin, which are included in this antimicrobial category. The second exception was observed with tetracycline, where the relationship between resistance genes and phenotypical resistance did not consistently align. This discrepancy in isolate 258.883 may be attributed to a nucleotide substitution at position 924 within the *tet*(A) gene (position 3,323, GenBank: AF534183.1), specifically transitioning from cytosine (C) to thymine (T). Because of this alteration, there is a shift in the protein composition from alanine (Ala) at position 118 to threonine (Thr). Although these are not the first *E. coli* isolates that harbor this gene mutation (89), to the authors knowledge our study is the first that associates this mutation in *tet*(A) to a failure in phenotypic response. Also, in more than 40% (4/9) of the isolates considered phenotypically resistant to amphenicols, no resistance gene associated with this antimicrobial category was found.

The phylogenetic tree (Figure 3) showed that canine isolates clustered together, except isolate 271.960 (ST 372), which was grouped with a human isolate (assembly reference OX637964.1) that belongs to ST131. ST 131 is one of the predominant sequence types within the ExPEC pathotype worldwide (71, 90). In fact, *E. coli* O25b:H4/ST131 was described as a prevalent clone in Spanish human population. In accordance with bibliography, this canine isolate was not associated with ESBL resistance and had a similar resistance profile to human strains (91, 92).

According to the virulence factors analyzed, most of the *E. coli* isolates found in urine samples were categorized in the ExPEC pathotype, as expected. The most frequently detected virulence factors were *papC* and *hlyA*, followed by *cnf1*. *Cnf1* prevalence in these isolates was similar to that found in isolates from both dogs and humans, while papC and *hlyA* prevalence were higher in this study (18, 93–97). However, most of the sampled populations in these studies include healthy animals, which could lower the prevalence of *E. coli* virulence factors. The prevalence of these three virulence factors were higher in dog isolates than in those found in humans (95, 98–100). Some of these factors were found in MGE (Table 5), which highlights their potential of spread to other strains.

Almost 20% of these ExPEC isolates displayed in combination with the three extraintestinal virulence-related genes analyzed (*papC*, *hlyA* and *cnf1*), likely due to the presence of a PAI (101). This type of virulence factors are frequently found in *E. coli* strains causing extraintestinal disease in both humans and dogs, being thus this animal species a possible reservoir for the ExPEC pathotype (102).

Apart from that there was one Stx2-positive isolate, that did not harbor any other virulence factor studied. Shiga toxin 2 is believed to be associated with the development of HUS (11) and is better produced when it is found in combination with other strains or bacteria (103). However, there are also some descriptions of Shiga toxin-producing *E. coli* isolates associated with UTI cases, and it has been proposed that Shiga toxins can bind to receptors from urinary bladder epithelial cells and damage them (104, 105). Additionally, an EPEC isolate was also detected. It is not the first time that an *eae*-positive isolate has been found among UTI-associated strains, although its frequency seems to be quite low as well (8, 106, 107). The role of this gene product (i.e., intimin) in UTI pathogenesis is not fully understood and its significance remains to be studied (8).

Taking into consideration that fecal *E. coli* population might have a relationship with UTI pathogenesis (108), it may be suggested that certain diarrheagenic pathotypes also have potential to cause UTI, although uncommon. However, the role of these strains in UTI development and the molecular and pathogenic causes behind it are still poorly understood, and more research in this field is needed in order to comprehend the mechanisms and epidemiological causes. Nevertheless, the ability of such strains to cause an extraintestinal infection in the host is not only dependent on their virulence-related genes but also on risk factors such as age or immunosuppression (106).

It is also important to note that a wide variety of extraintestinal-associated virulence traits has been described in the literature. Thus, apart from these virulence genes typical of diarrheagenic strains, these two isolates might be also harboring some other extraintestinal virulence factors different from those analyzed in this study. In this regard, some *E. coli* strains have been recently classified as hybrids for harboring virulence factors usually associated with various pathotypes, e.g., STEC/UPEC strains (109). The genome plasticity of this microorganism promotes the exchange and combination of both intestinal and extraintestinal virulence determinants, resulting in an heteropathogenic potential (106, 107). The possible emergence of hybrid pathotypes not only in humans but also in animals should therefore be surveilled.

The finding of eight isolates (15.38%) considered neither intestinal nor extraintestinal pathogenic isolates could be explained by the fact that only a selection of virulence factors was tested. Thus, these *E. coli* might harbor other different virulence-related genes not analyzed in this study. However, another explanation could be that the causal agent for UTI in these dogs was different from *E. coli*, or even a non-infectious cause. It is also worth noting that the detection of virulence factor genes does not mean that they are phenotypically expressed, so the severity of the disease could not be only assessed with this information. Nevertheless, it is known that the severity of the disease is not caused by a single virulence factor but a combination of them (110, 111).

The most commonly isolated serotype in this study was O5H20. In this regard, O5:H(−) has been associated with STEC strains, and Shiga toxins have been also described in *E. coli* strains causing UTI (112, 113). However, these isolates did not harbor any Shiga toxin gene. The rest of *E. coli* serotypes are distributed along different STs and antibiotic resistance patterns, showing a heterogenic distribution.

Interestingly, low antibiotic resistance patterns were linked to a higher number of virulence factors. There is some literature (114, 115) that suggests a positive relationship between virulence factors and MDR. However, in isolates from this study only *cnf1* carriage showed a significant association with MDR, while a high virulence factor carriage was associated with low resistance profiles. The reason for this mechanism is still unclear, but it is hypothesized that the acquisition of MDR is “sacrificed” in exchange for virulence factors, or that the low presence of virulence factors facilitates the acquisition of antibiotic resistance (116, 117). When analyzing correlation between all virulence factors found and the presence of antibiotic resistance genes in whole genome sequenced isolates, the relationship was non-significant (*p* = 0.14). However, there was a bias in selection of isolates, as only the more resistant ones were chosen.

## 5 Conclusion

Based on these data, a very high percentage of *E. coli* isolates found in urine samples from dogs suffering from UTI was considered MDR, the majority of them being classified as ExPEC. Phenotypic antimicrobial resistance to first-lines agents recommended in UTI management was also frequently observed, which could be associated with a treatment failure. Furthermore, several antimicrobial resistance genes, some of them contained in MGE, were identified in the genome of selected resistant isolates. The use of WGS could identify some of the genetic mechanisms underlying antimicrobial resistance, although there were a few discordances between phenotypic resistance and genes found. Combining both phenotypic and genetic data enhances our understanding of antibiotic resistance and improves treatment selection efficiency.

Overall, these findings are of concern for both animal and public health, since dogs could act as reservoirs of MDR pathogenic *E. coli* and contribute to the spread of antimicrobial resistance. Surveillance of antimicrobial resistance and revision of therapeutic guidelines should be therefore continuously addressed in clinical veterinary settings.

## Data availability statement

The original contributions presented in the study are publicly available. This data can be found at: https://www.ncbi.nlm.nih.gov/bioproject; PRJNA1031085.

## Ethics statement

Ethical approval was not required for the studies involving animals in accordance with the local legislation and institutional requirements because samples were collected as part of the daily activity of private veterinary practitioners. Written informed consent was obtained from the owners for the participation of their animals in this study.

## Author contributions

AA-F: Data curation, Formal analysis, Investigation, Writing – original draft, Writing – review & editing. ES: Data curation, Formal analysis, Supervision, Writing – original draft, Writing – review & editing. AO: Investigation, Writing – original draft. IM-B: Formal analysis, Supervision, Writing – review & editing. BM: Resources, Supervision, Writing – review & editing. MM: Conceptualization, Resources, Supervision. RB: Conceptualization, Funding acquisition, Resources, Supervision, Writing – review & editing.
